# Berberine Inhibits the Inflammatory Response Induced by *Staphylococcus aureus* Isolated from Atopic Eczema Patients via the TNF-α/Inflammation/RAGE Pathways

**DOI:** 10.3390/cells13191639

**Published:** 2024-10-01

**Authors:** Anish R. Maskey, Daniel Kopulos, Matthew Kwan, Niradiz Reyes, Christian Figueroa, Xian Mo, Nang Yang, Raj Tiwari, Jan Geliebter, Xiu-Min Li

**Affiliations:** 1 Department of Pathology, Microbiology & Immunology, New York Medical College, Valhalla, NY 10595, USA; amaskey@nymc.edu (A.R.M.); dkopulos@student.touro.edu (D.K.); mattbradk@yahoo.ca (M.K.); niradiz@gmail.com (N.R.); christian.figueroa@richmond.edu (C.F.); xianmo@gird.cn (X.M.); raj_tiwari@nymc.edu (R.T.); 2 Genetics and Molecular Biology Research Group, School of Medicine, University of Cartagena, Cartagena 130001, Colombia; 3 Department of Biology, University of Richmond, Richmond, VA 23173, USA; 4 Department of Allergy and Clinical Immunology, Guangzhou Institute of Respiratory Health, Guangzhou 510120, China; 5 General Nutraceutical Technology, Elmsford, NY 10523, USA; nan.yang@gnt-us.com; 6 Department of Otolaryngology, New York Medical College, Valhalla, NY 10595, USA; 7 Department of Dermatology, New York Medical College, Valhalla, NY 10595, USA

**Keywords:** atopic dermatitis, *S. aureus*, berberine, inflammation, TNF-α

## Abstract

Atopic eczema patients exhibit high levels of *Staphylococcus aureus* (*S. aureus*) skin colonization. *S. aureus* can stimulate macrophages and the expression of proinflammatory cytokines. Berberine (BBR), an alkaloid, attenuates *S. aureus* toxin production. This study investigated if BBR suppressed bacterial growth and inflammatory response induced by eczema-patient-derived *S. aureus* using murine macrophage (RAW 264.7) and human monocyte cell lines (U937). RAW 264.7 and U937 were treated with BBR at different concentrations and stimulated with heat-killed *S. aureus* (ATCC #33591) or *S. aureus* derived from severe eczema patients (EC01–EC10), who were undergoing topical steroid withdrawal, for 24 h. TNF-α protein levels were determined by ELISA, gene expression by qRT-PCR, cell cytotoxicity by trypan blue excursion, and reactive oxygen species (ROS) levels by fluorometric assay. BBR showed a bacteriostatic effect in *S. aureus* (ATCC strain #33591 and clinical isolates (EC01–EC10) and suppressed TNF-α production in RAW 264.7 and U937 cells exposed to heat-killed *S. aureus* (ATCC and clinical isolates) dose-dependently without any cell cytotoxicity. BBR (20 µg/mL) suppressed >90% of TNF-α production (*p* < 0.001), downregulated genes involved in inflammatory pathways, and inhibited *S. aureus* ROS production in U937 and RAW 264.7 cells (*p* < 0.01). BBR suppresses *S. aureus*-induced inflammation via inhibition of TNF-α release, ROS production, and expression of key genes involved in the inflammatory pathway.

## 1. Introduction

Atopic eczema is a chronic inflammatory condition of the skin characterized clinically by flares of dry, red, itchy skin lesions [1]. During skin flares, the normal microbial diversity of the skin is severely diminished allowing pathogenic bacteria such as *S. aureus* to colonize and proliferate [2]. Once present, *S. aureus* further inhibits the growth of commensals, exacerbating the disease and symptoms. Ten to thirty percent of *S. aureus* isolated from patients with atopic eczema are methicillin-resistant (MRSA) [3]. Mouse models of atopic dermatitis (AD) have shown that *S. aureus* may promote or exacerbate skin lesions [4,5]. At the site of infection, *S. aureus* can secrete a battery of toxins, including enterotoxins, which has been shown to activate immune cells and induce inflammatory responses to further exacerbate eczema [6,7].

TNF-α plays a critical role in initiating the inflammatory process and contributes to disease pathogenesis by directly inducing inflammatory gene expression or indirectly inducing cell death, leading to the instigation of additional inflammatory immune reactions [8]. TNF-α also promotes inflammation by triggering lytic forms of death, such as apoptosis-driven secondary necrosis, pyroptosis, and necroptosis which release intracellular factors that activate proinflammatory gene expression in bystander cells [8]. One of the first responses to infection by *S. aureus* is the activation of mononuclear phagocytes [9]. Professional phagocytes such as macrophages can recognize the pathogen and induce an inflammatory response by producing proinflammatory cytokines. These cells recognize bacterial components through Toll-like receptors (TLRs) and activate the signaling pathways via the MYD88 innate immune signal transduction adaptor and nuclear factor kappa-light-chain-enhancer of activated B cells (NF-κB) to upregulate the transcription of proinflammatory cytokines, *TNF-α*, *IL-1β*, and *IL-6*, which are key players in the onset of septic shock [10]. In combination with releasing additional chemokines (e.g., CXCL8), TNF-α induces the expression of adhesion molecules on endothelial cells which facilitates the recruitment of leukocytes and the release of additional inflammatory mediators [10,11]. In addition, ROS act as both signaling molecules and mediators of inflammation [12]. Although it is evident that *S. aureus* stimulates the production of ROS [13], the physiological contribution to the mechanisms of inflammation and tissue damage remains unclear. Numerous anti-TNF-α biologic therapies have been developed over the years to mitigate dysregulated inflammatory processes by targeting and neutralizing TNF-α [14]. However, present evidence suggests there is an increased risk of infection and malignancies associated with anti-TNF-α treatment [15].

BBR, an isoquinoline compound extracted from several plant species, has a long history of use in traditional Chinese medicine (TCM) due to its various health benefits, including anti-inflammatory and antimicrobial activities [16,17,18]. Notably, BBR and its derivatives have been shown to have therapeutic effects on intestinal, pulmonary, skin, and bone inflammatory disorders [16]. It suppresses LPS-induced proinflammatory genes including *IL-1β*, *IL-6*, and *MCP-1* by activating AMPK signaling in macrophages [19]. Furthermore, the AMPK and NF-κB signaling pathways, which are involved in controlling inflammation, are key targets for the anti-inflammatory activity of BBR [19,20]. In addition, BBR has also been extensively studied for its antimicrobial effects. In LPS-induced inflammatory responses it has consistently been shown to inhibit proinflammatory cytokines such as TNF-α [21]. Furthermore, in previous studies, BBR has demonstrated an antimicrobial effect on *S. aureus* by inhibiting its bacterial growth in a dose-dependent manner [17]. Due to BBR’s anti-inflammatory nature, this study aimed to investigate BBR’s mechanisms in attenuating the inflammatory response induced by a commercially available MRSA strain and clinical *S. aureus* isolates cultured from the skin of severe eczema patients, who were undergoing topical steroid withdrawal (TSW) syndrome. The investigation was conducted in vitro using murine macrophage (RAW 264.7) and human monocyte (U937) cell lines.

## 2. Materials and Methods

### 2.1. High-Performance Liquid Chromatography (HPLC) Analysis of BBR

BBR was obtained from Xi’an SaiYang Biotechnology, LLC (Xi’an, China). BBR was analyzed using an HPLC system which consists of a Waters 2690 separation module coupled with a 2996 PDA detector (Waters, Milford, MA, USA). BBR was dissolved in methanol at 50 µg/mL, and 10 µL of the sample solution was loaded on a ZORBAX SB-C18 (4.6 × 150 mm, 5 µm) column (Agilent, Santa Clara, CA, USA) for separation. Then, 0.1% formic acid solution was used as mobile phase A and HPLC grade acetonitrile (Fisher Scientific, NJ, USA) was used as mobile phase B. The flow rate was set as 1 mL/min. The gradient elution process was as follows: from 10% mobile phase B to 100% in 10 min and maintained at 100% mobile phase B for 3 min. The chromatographic data were acquired at a wavelength of 264 nm and processed using Waters Empower 3 software (Figure S1).

### 2.2. Isolation and Identification of S. aureus from Eczema Patients

Skin swabs were taken from topical steroid withdrawal (TSW) syndrome patients (*n* = 8) visiting the Center for Integrative Health and Acupuncture at the Otolaryngology Department, Westchester Medical Center (WMC), according to the protocol of the Institutional Review Board at NYMC (IRB protocol #12756). The swab was rolled onto a mannitol salt agar (MSA) plate (Thermofisher Scientific, MA, USA), covering the entire surface of the plate. The plate was incubated in a CO_2_ incubator at 37 °C for 24 h. Single isolated colonies were obtained, and these colonies were further streaked onto nutrient agar (NA) (Thermofisher Scientific, MA, USA) and incubated for 24 h. Two or three well-isolated single colonies were emulsified in a DNA/RNA shield collection tube (Zymo Research Corporation, CA, USA) and were used for sequencing analysis to identify *Staphylococcus* strains. Eight different strains, each from a different patient, were identified, and were coded as EC01, EC02, EC03, EC04, EC06, EC07, EC09, and EC10, respectively.

### 2.3. Bacteria Preparation and Cell Stimulation

*S. aureus* (#33591, ATCC, VA) and all the clinical strains (EC01–EC10) were streaked onto a nutrient agar plate and incubated at 35–37 °C 24 h. Several isolated single colonies were selected and inoculated into the nutrient broth (NB) and incubated for 4 h in a 37 °C shaker at 225 rpm. The turbidity was adjusted to obtain 0.5 MacFarland standard (1.2 × 10^8^ CFU/mL) by measuring O.D. values at 625 nm using a spectrophotometer. The O.D. value was adjusted to 0.08–0.1, equivalent to 0.5 MacFarland standard. The bacteria were heat killed by incubating at 65 °C for 1 h. The multiplicity of infectivity (MOI) was determined based on the number of cells used for the different experimental conditions and the bacterial suspension was added to respective experimental wells.

### 2.4. Minimum Inhibitory Concentration

Serial dilutions of BBR at different concentrations (512, 256, 128, 64, 32 µg/mL) were prepared in nutrient broth. Several well-isolated colonies of clinical strains *S. aureus* (EC01–EC10) grown in NA were picked and re-suspended in NB. The concentration of the bacteria was adjusted to 0.5 MacFarland standard. Then, a 1:100 dilution of 0.5 MacFarland was prepared and equal volumes of different concentrations of BBR and dilute bacterial suspension were mixed in a separate tube and incubated for 18–22 h in a 225-rpm shaker at 37 °C. After 18–22 h of incubation, minimum inhibitory concentration (MIC) was determined by measuring the O.D. values of each tube at 625 nm. The concentration of BBR at which no visible growth occurred was determined to be the MIC.

### 2.5. Cell Culture, TNF-α Measurement, and Cell Viability Assessment

RAW 264.7 cells (ATCC, VA) were cultured in a growth medium containing DMEM (Gibco, NY, USA) supplemented with 10% fetal bovine serum (FBS) (BioWest, FL, USA) and 1% penicillin/streptomycin (Corning, VA, USA). Cells were cultured for 2–3 days until 75% confluency was observed. RAW 264.7 cells were plated onto a 48-well culture plate at a concentration of 2 × 10^5^ cells/mL and allowed to attach to the surface of the plate for 1–2 h. BBR at concentrations of 2.5, 5, 10, and 20 µg/mL was added to the respective wells immediately followed by stimulation with heat-killed *S. aureus* (HKS) at a multiplicity of infection (MOI) of 2:1. The cells containing different concentration of BBR and HKS were incubated for 24 h, after which cell culture supernatant was harvested and TNF-α levels were determined by ELISA as per the manufacturer’s instructions. Cell viability was evaluated using trypan blue exclusion. Similarly, U937 cells (ATCC, VA, USA) were cultured in ATCC-formulated RPMI 1640 (Gibco, NY, USA) medium containing 10% FBS and 1% penicillin/streptomycin. Cells were cultured for 2–3 days until a 75% confluence was observed. The cells were then used for the experiment. Briefly, cell concentration was adjusted to 2 × 10^6^/mL, of which 250 µL/well was plated on a 48-well cell culture plate. Then, 250 μL of BBR at different concentrations (2.5, 5, 10, 20 µg/mL) was added to each well to make a total volume of 500 µL per well. The BBR-treated cells were then stimulated with ATCC and clinical strains of HKS at MOI of 2:1. The cells were incubated for 24 h, after which cell supernatants were harvested and TNF-α levels were determined by ELISA (BD Biosciences, CA, USA) following the manufacturer’s protocol. Cell cytotoxicity was evaluated by trypan blue exclusion (Thermo Fisher Scientific, MA, USA).

### 2.6. qRT-PCR Analysis

U937 cells (2 × 10^6^/mL) were added to a 6-well plate along with BBR (20 μg/mL), immediately followed by stimulation with the ATCC strain HKS at MOI of 2:1. Cells were incubated at 37 °C in 5% CO_2_ for 24 h. The cell pellet was harvested, and RNA was extracted by adding RLT buffer following the extraction protocol of the All-prep DNA/RNA Mini Kit (QIAGEN, MD, USA) according to the manufacturer’s instructions. Total RNA was subjected to reverse transcription using a Revert Aid RT Kit (Thermofisher Scientific, MA, USA) and expression levels we determined by qRT-PCR using SYBR^®^ Green PCR Master Mix (Thermo Fisher Scientific, MA, USA). The human primer sequence in the study is listed in the Supplementary Materials (Table S1).

### 2.7. Reactive Oxygen Species

Intracellular reactive oxygen species (ROS) levels were measured using the DCFDA/H2DCFDA-Cellular ROS assay kit (Abcam, MA, USA) as per the manufacturer’s protocol. Briefly, RAW 264.7 cells (2.5 × 10^4^) were serum starved for 24 h and seeded on a dark, clear-bottom 96-well microplate overnight to allow the cells to adhere. After overnight incubation, the cells were washed with PBS twice and then pre-treated with BBR (20 µg/mL) for 30 min after which the cells were stimulated with the ATCC strain HKS (MOI, 2:1). Twenty-four hours later, 20 μM dichlorofluorescein diacetate (DCFDA, Abcam, MA, USA) was overlayed on top of the treated cells for 45 min at 37 °C in the dark. After this, the plate was read in an endpoint setting using a fluorescent reader (SpectraMax ID, Molecular Devices, CA, USA) with excitation and emission wavelengths of 485 and 535 nm, respectively. The data are represented as relative fluorescence unit (RFU). In a similar experiment, U937 cells were cultured with BBR (20 µg/mL) and stimulated with the ATCC strain HKS at MOI of 2:1. The cells were incubated overnight at 37 °C. After this, the cells were washed twice with PBS. ROS levels were measured as described previously.

### 2.8. Statistical Analysis

All statistical analyses were conducted using GraphPad Prism 9 (San Diego, CA, USA). One-way analysis of variance (ANOVA) was employed, followed by Bonferroni correction for all pairwise comparisons. For skewed data, differences between groups were assessed using one-way ANOVA on ranks followed by Dunn’s test. *p*-value calculations were two-tailed, with a *p*-value < 0.05 considered statistically significant.

## 3. Results

### 3.1. BBR Inhibited Bacterial Growth of S. aureus #33591 and Clinical Isolates

**Figure 1:**
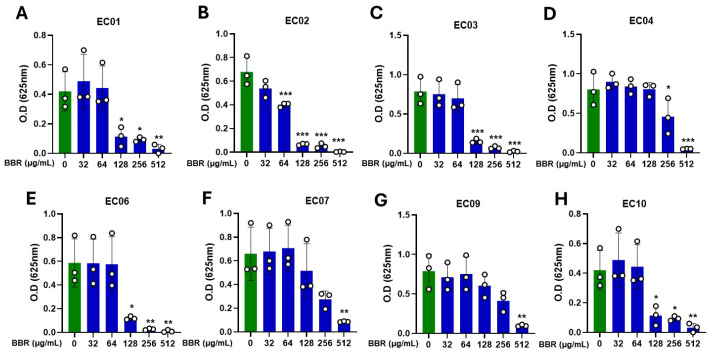
Inhibitory effect of BBR on clinical *S. aureus* isolates. Clinical *S. aureus* strains (EC01–EC10) were cultured in the presence of BBR at different concentrations (32 μg/mL, 64 μg/mL, 128 μg/mL, 256 μg/mL, and 512 μg/mL) for 18–20 h. MIC was determined by measuring absorbance at 625 nm. BBR showed dose-dependent inhibition of bacteria growth in clinical strains. (**A**) EC01; (**B**) EC02; (**C**) EC03; (**D**) EC04; (**E**) EC06; (**F**) EC07; (**G**) EC0; and (**H**) EC10. Green–without BBR; Blue–with BBR. Data represent mean ± SD. (*N* = 3 replicates; * *p* > 0.05; ** *p* > 0.01; *** *p* < 0.001 vs. 0).

BBR showed a dose-dependent inhibition of bacteria growth of clinical *S. aureus* strains (EC01–EC10). The MIC was 128 µg/mL for EC02, 256 µg/mL for strains EC01, EC03, EC06, and EC10, and 512 µg/mL for EC04, EC07, and EC09 (Figure 1A–H). Furthermore, BBR also showed a similar dose-dependent inhibition effect in ATCC #33591 *S. aureus*. The MIC was found to be 128 µg/mL (Figure S2). These results show that BBR has a bacteriostatic effect on clinically identified and ATCC *S. aureus* strains.

### 3.2. BBR Inhibited TNF-α Production by RAW 264.7 and U937 Cells Following S. aureus #33591 Stimulation

**Figure 2:**
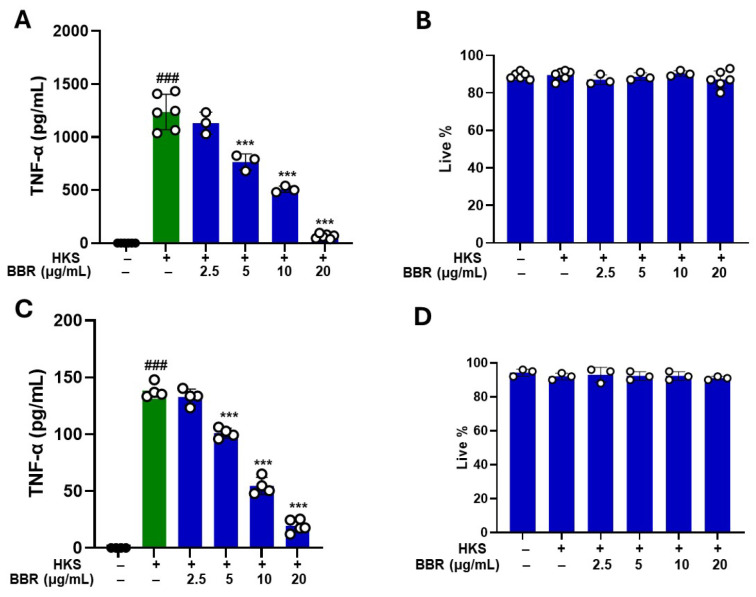
Dose-dependent effect of BBR on ATCC strain heat-killed *S. aureus.* RAW 264.7 cells were cultured in the presence and absence of ATCC strain (#33591) HKS (MOI, 1:2) and BBR at different concentrations (2.5 μg/mL, 5 μg/mL, 10 μg/mL, and 20 μg/mL) for 24 h. (**A**) BBR pre-treatment showed a dose-dependent inhibition of TNF-α levels following HKS stimulation. (**B**) Cell viability measured by trypan blue exclusion showed no cell cytotoxicity across all groups. (**C**,**D**) Similar results were observed in U937 following BBR treatment. (**A**,**C**); Green- without BBR; Blue- with BBR). Data represent mean ± SD. (*N* = 3–6 replicates; ^###^ *p* < 0.001 vs. untreated; *** *p* < 0.001 vs. HKS).

To determine the effect of BBR treatment on TNF-α production, a mouse macrophage cell line (RAW 264.7 cells) was cultured with different concentrations of BBR, followed by stimulation with HKS (ATCC #33591) at MOI 2:1. BBR showed a dose-dependent inhibition of TNF-α production, with 20 µg/mL completely inhibiting the ability of RAW 264.7 cells to produce TNF-α (*p* > 0.001) as compared to untreated control (Figure 2A). This inhibition was not associated with any cell cytotoxicity (Figure 2B). We then determined the effect of BBR on the human monocyte cell line (U937) under similar conditions. U937 cells were cultured with BBR at different concentrations (2.5, 5, 10, 20 µg/mL) followed by stimulation with HKS (ATCC #33591) at MOI 2:1. Similar results were found, with a robust increase in levels of TNF-α production by U937 cells following HKS stimulation (*p* > 0.001), followed by dose-dependent BBR response with 20 µg/mL inhibiting >90% TNF-α production as compared to untreated control (Figure 2C). This inhibition was not associated with any cellular cytotoxicity (Figure 2D).

### 3.3. BBR Inhibited TNF-α Production Following Heat-killed Clinical Strain S. aureus Stimulation

**Figure 3:**
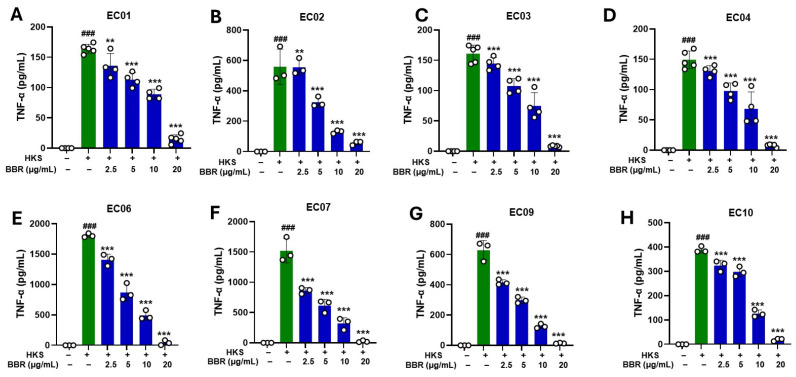
Dose-dependent effect of BBR on clinical isolates of heat-killed *S. aureus.* U937 cells were cultured in the presence and absence of clinical isolates of HKS (1 × 10^6^ CFU/mL) and BBR at different concentrations. BBR treatment (2.5 µg/mL, 5 μg/mL, 10 μg/mL, and 20 μg/mL) showed a dose-dependent inhibition of TNF-α production in the presence of heat-killed clinical *S. aureus* isolates (**A**–**H**) EC01, EC02, EC03, EC04, EC06, EC07, EC09, EC10. Green- without BBR; Blue- with BBR. TNF-α levels were measured by ELISA. Data represent mean ± SD. (*N* = 3–5 replicates; ^###^ *p* < 0.001 vs. untreated; ** *p* < 0.01; *** *p* < 0.001 vs. HKS).

Since human U937 monocytes showed consistent results as in murine macrophage RAW 264.7 cells in response to HKS stimulation and BBR treatment, we next focused on U937 cells to determine BBR inhibitory effects on TNF-α production following stimulation with heat-killed clinical *S. aureus* isolates. Strains EC06 (1808 ± 63.5 pg/mL) and EC07 (1521 ± 83.83 pg/mL) had the highest TNF-α stimulating ability, EC09 (628.6 ± 23.68 pg/mL), EC02 (559.1 ± 44.19 pg/mL), and EC10 (389.3 ± 12.08 pg/mL) had moderate TNF-α stimulation ability, whereas EC01 (164.2 ± 7.13 pg/mL), EC03 (160.9 ± 8.25 pg/mL), and EC04 (149.3 ± 9.27 pg/mL) showed the lowest level of TNF-α production (Figure 3A–H).

**Figure 4:**
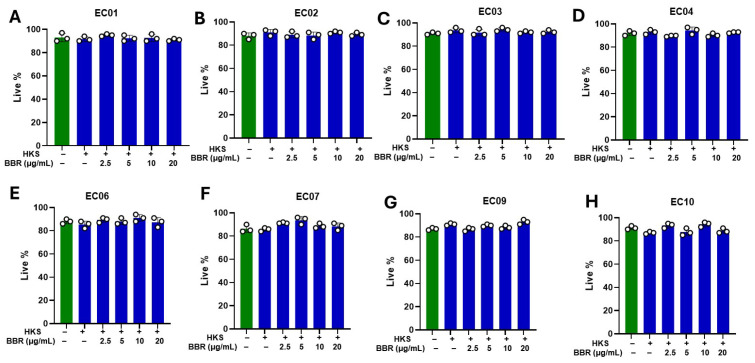
No cell cytotoxicity was observed with BBR, and heat-killed *S. aureus*. U937 cells were cultured in the presence and absence of clinical isolates of HKS (1 × 10^6^ CFU/mL) and BBR at different concentrations. BBR treatment (2.5 µg/mL, 5 μg/mL, 10 μg/mL, and 20 μg/mL) showed no cell cytotoxicity at any concentration for clinical strains (**A**–**H**) EC01, EC02, EC03, EC04, EC06, EC07, EC09, EC10. Green- without BBR & HKS; Blue- with BBR & HKS. Cell cytotoxicity was evaluated by trypan blue exclusion. Data represent mean ± SD. (*N* = 3 replicates).

We found that a dose as low as 2.5 μg/mL of BBR significantly reduced TNF-α production. These results were consistent across all clinical strains (Figure 3A–H). We found greater inhibition with higher BBR doses and achieved 90–100% inhibition at 20 μg/mL (Figure 3A–H; *p* < 0.001). Furthermore, BBR showed no cell cytotoxicity following *S. aureus* stimulation of multiple clinical strains (Figure 4A–H). Taken together, these results indicate that the clinical strains isolated from human eczema skin have the potential to stimulate TNF-α production by U937 cells and BBR treatment inhibits this ability without any cell cytotoxicity. Furthermore, all strains are different from each other and show variable TNF-α levels following stimulation.

### 3.4. BBR Inhibited Genes Associated with TNF-α, AGE-RAGE, and Inflammatory Signaling Pathways

**Figure 5:**
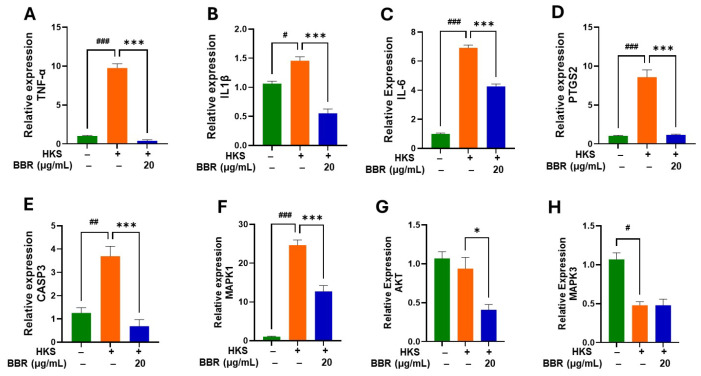
Effect of BBR on gene expression associated with TNF, AGE-RAGE, and inflammatory pathways. U937 cells were cultured in the presence in BBR (20 μg/mL) and ATCC (#33591) HKS (MOI, 2:1) for 24 h. Cells were harvested, and gene expression analysis was performed by qRT-PCR. There was a significant decrease in expression of (**A**) TNF-α; (**B**) IL-1β, (**C**) IL-6; (**D**) PTGS2; (**E**) CASP3; (**F**) MAPK1; (**G**) AKT; (**H**) MAPK3 in the presence of BBR. Green- without BBR & HKS; Orange- with HKS & without BBR; Blue- With HKS & BBR. Data represent mean ± SD. (*N* = triplicate culture. ^#^ *p* < 0.05; ^##^ *p* < 0.01; ^###^ *p* < 0.001 vs. untreated; * *p* < 0.05; *** *p* < 0.001 vs. HKS).

To determine the mechanism of action of BBR on TNF-α inhibition we studied key target molecules of relevant pathways associated with BBR inhibition of inflammation [22]. The pathways which were highly relevant to inflammation included TNF-α, AGE-RAGE, and inflammatory pathways (Table 1). We found 10 genes associated with TNF-α pathways, including *TNF*, *IL-1β*, *IL-6*, *AKT1*, *MAPK1*, *MAPK3*, *CASP3*, *PTGS2*, *MAPK14*, and *CREB1*. Similarly, nine genes were associated with AGE-RAGE pathways, including *TNF*, *IL-1β*, *IL-6*, *AKT1*, *MAPK1*, *MAPK3*, *CASP3*, *PTGS2*, *MAPK14*, and *CCND1*, and thirteen genes were associated with inflammatory pathways, including *TNF*, *IL-1β*, *IL-6*, *AKT1*, *MAPK1*, *MAPK3*, *CASP3*, *PTGS2*, *MAPK14*, *CCND1*, *CREB1*, *CDK6*, and *HIF1A* (Table 1). Nine genes were common in all three pathways. In addition, *CCND1* was common in TNF and AGE-RAGE pathways and *CREB1* was common in TNF and inflammatory pathways. In order to validate if BBR had any effect on the expression of genes associated with the three highly relevant pathways we measured the expression of the top eight genes following ATCC #33591 HKS stimulation using qRT-PCR (Figure 5). We found significant inhibition of expression of (A) *TNF-α* (*p* < 0.001); (B) *IL-1β* (*p* < 0.001); (C) *IL-6* (*p* < 0.001); (D) *PTGS2* (*p* < 0.001); (E) *CASP3* (*p* < 0.01); (F) *MAPK1* (*p* < 0.001); and (G) *AKT* (*p* < 0.05) in the presence of BBR (20 µg/mL) as compared to untreated control. There was no significant difference in the expression levels of (H) *MAPK3* between BBR-treated and untreated groups. Overall, these results show that BBR has an anti-inflammatory effect, and its anti-TNF activity is associated with inhibition of expression of key genes and targets associated with the highly relevant inflammatory pathways.

### 3.5. BBR Inhibited ROS Production Following HKS Stimulation

**Figure 6:**
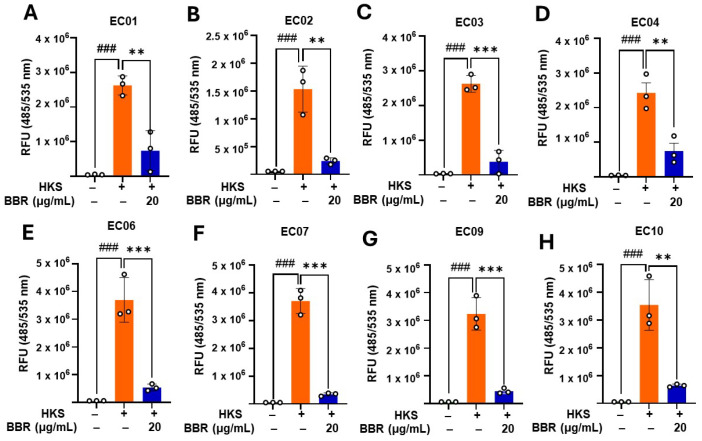
Effect of BBR on ROS production: Intracellular reactive oxygen species (ROS) levels were measured using the DCFDA/H2DCFDA-Cellular ROS assay kit (Abcam, MA) as per the manufacturer’s instructions. U937 pre-treated with BBR (20 μg/mL) showed reduced ROS production as compared to untreated cells stimulated with clinical *S. aureus* strains. (**A**) EC01; (**B**) EC02; (**C**) EC03; (**D**) EC04; (**E**) EC06; (**F**) EC07, (**G**) EC09; and (**H**) EC10. Green- without BBR & HKS; Orange- with HKS & without BBR; Blue- With HKS & BBR. Data represent mean ± SD. (*N* = triplicate culture. ^###^ *p* < 0.001 vs. untreated; ** *p* < 0.01; *** *p* < 0.001 vs. HKS).

Since the most potent TNF-α inhibition effect was observed for BBR (20 µg/mL), we further evaluated the effect of BBR (20 µg/mL) on ROS production in U937 cells. Our results show that BBR (20 µg/mL) significantly inhibited ATCC #33591 HKS-stimulated ROS production by U937 cells (*p* < 0.01) (Figure S3A). Furthermore, to extend this effect, we evaluated the effect of BBR in inhibiting ROS production in RAW 264.7 cells. The results indicate significant inhibition of ROS with BBR treatment (Figure S3B). As all the clinically isolated strains showed potent TNF-α stimulating activity, we wanted to further study the effect of BBR (20 µg/mL) on ROS production by the clinically isolated strains. Remarkably, BBR (20 µg/mL) reduced the ability of all clinical HKS strains EC01, EC02, EC03, EC04, EC06, EC07, EC09, and EC10 to induce ROS production following U937 cell stimulation (Figure 6A-H). Overall, these results highlighted the in vitro protective effect of BBR (20 µg/mL) in preventing detrimental effects of ROS stimulated by *S. aureus*.

## 4. Discussion

In this study, we demonstrate that BBR significantly inhibited TNF-α production in both mouse macrophage and human monocyte cell lines (RAW 264.7 and U937). This was observed after stimulation with HKS ATCC #33591 and clinically isolated *S. aureus* strains. Remarkably, this inhibition was not associated with any cell cytotoxicity. The consistent inhibition of TNF-α production by BBR across *S. aureus* strains identified from multiple individuals with atopic eczema highlights its potential as a therapeutic option for *S. aureus*-exacerbated atopic eczema. These finding are particularly relevant to eczema patients who frequently experience *S. aureus* colonization and often seek new treatments due to the limited therapies. Our results demonstrated that the anti-TNF-α activity of BBR is associated with the modulation of key genes associated with three highly relevant pathways, including TNF-α, AGE-RAGE, and inflammatory pathways. BBR remarkably inhibited the expression of TNF-α, IL-1β, IL-6, PTGS2, CASP3, and MAPK1 following *S. aureus* stimulation. TNF-α is a central cytokine that orchestrates an inflammatory response, induces cell death, and instigates disease development [8]. Similarly, IL-1β promotes the recruitment of inflammatory cells to the site of infection and creates a proinflammatory environment favorable for other inflammatory cytokines to act [23,24]. IL-6 is a proinflammatory cytokine produced in the body whenever there is inflammation, either acute or chronic [25]. The combined action of all these cytokines leads to severe inflammation ultimately progressing toward cytokine storm. Previously, BBR has demonstrated anti-inflammatory properties, such as inhibiting key cytokines and transcription factors like NF-κB and AP-1 [22,26,27] and suppressing the release of TNF-α and IL-6 [28]. Our study further supports these findings and identifies key pathways and targets using in silico computational modeling. We validated some of the important targets using in vitro experimental approaches. Our results showed BBR reduced the expression of many inflammatory genes like TNF-α, IL-1β, IL-6, PTGS, MAPK1, CASP3, and AKT. Additionally, due to its anti-inflammatory and antiviral effects, BBR has also been used as a potential COVID-19 treatment agent [28].

In addition, our results indicated that BBR significantly decreased the ability of RAW 264.7 and U937 cells to produce ROS following stimulation with different strains of *S. aureus* isolated from atopic eczema patients. ROS are potent triggers of oxidative stress and initiate inflammation, tissue injury, and cellular damage [12]. This becomes critical in patients with atopic eczema as the majority of these patients present with severe, inflamed skin lesions with open wounds. Therefore, it is important to understand how ROS contribute to tissue damage and how BBR mitigates potent ROS activity in the context of atopic eczema. Previous studies have shown that BBR prevents ROS production by neutrophils following phorbol 12-myristate 13-acetate (PMA) stimulation [29]. To date, this is the first study to report the inhibitory effect BBR on ROS production following *S. aureus* colonization. This effect could be due to the inhibitory effect of BBR on TLRs and needs to be further explored. It is reported that colonization of *S. aureus* is found in 70% of atopic eczema skin lesions [30]. *S. aureus* colonization in AD reduces commensal skin bacteria that promote the secretion of antimicrobial peptides (AMPs) to protect skin from invading pathogens [31]. This study provides a basis on how *S. aureus* could potentially stimulate ROS production by monocytes to contribute to tissue damage. Further studies using *S. aureus*-induced atopic in vivo eczema models are necessary to confirm these findings.

Current research on *S. aureus* infections faces significant challenges and gaps that need to be addressed. One of the primary concerns is the rise of antibiotic-resistant strains, particularly MRSA, which limits treatment options and complicates patient care. There is a pressing need for the development of new antibiotics effective against these resistant strains, but the pipeline for novel antibiotics remains insufficient. Another critical issue is the formation of *S. aureus* biofilms, which pose a substantial obstacle to treatment efficacy. Biofilms create a protective environment that enhances bacterial resistance to both antibiotics and the immune system, leading to persistent infections that are difficult to eradicate. Finding strategies to disrupt or prevent biofilm formation is crucial for improving outcomes in *S. aureus* infections. In this context, small molecules like BBR have emerged as potential candidates for inhibiting the growth and virulence of *S. aureus*. BBR, derived from plants, has shown antimicrobial properties against various pathogens, including *S. aureus*. Studies indicate that BBR can disrupt bacterial cell membranes, inhibit biofilm formation, and reduce bacterial virulence factors [17,32]. Moreover, BBR possesses anti-inflammatory properties, which may help mitigate the excessive inflammatory response triggered by *S. aureus* infections [22]. Additionally, BBR has an inhibitory effect on TLRs, specifically TLR-4 in the context of inflammation [33,34,35]. However, it remains unknown whether BBR’s anti-inflammatory effects on clinical *S. aureus* strains are via inhibition of TLRs and they need to be considered in future studies. Similarly, there are many toxins associated with *S. aureus* and how BBR mitigates these toxins needs further exploration. Likewise, there remain gaps in the literature regarding the optimal use of BBR. It is essential to conduct more studies to understand the specific molecular mechanisms through which BBR acts against *S. aureus* and to evaluate its effectiveness in clinical settings. Additionally, investigating BBR’s potential in combination therapies with existing antibiotics could open new avenues for combating antibiotic-resistant *S. aureus* infections.

## 5. Conclusions

Overall, this broad inhibition of bacterial growth and inflammatory responses emphasizes BBR’s potential as a comprehensive anti-inflammatory agent in *S. aureus*-exacerbated atopic eczema. This study sheds light on BBR’s anti-inflammatory mechanism by inhibiting the expression of key inflammatory genes such as TNF, IL-1β, IL-6, CASP3, and PTGS2 which are important genes associated with highly relevant TNF, AGE/RAGE, and inflammatory pathways. Furthermore, the reduction in ROS production by BBR leads us to conclude that BBR could be a promising therapeutic candidate for managing *S. aureus*-mediated eczema. This research further paves the way for innovative treatment strategies aimed at improving outcomes for patients suffering from inflammatory conditions and persistent bacterial infections.

## Figures and Tables

**Table 1:** Pathways and gene associated with BBR effect on TNF-α production.

Pathways	Genes												
TNF-α	*TNF*	*IL1* *β*	*IL6*	*AKT1*	*MAPK1*	*MAPK3*	*CASP3*	*PTGS2*	*MAPK14*		*CREB1*		
AGE-RAGE	*TNF*	*IL1* *β*	*IL6*	*AKT1*	*MAPK1*	*MAPK3*	*CASP3*	*PTGS2*	*MAPK14*	*CCND1*			
Inflammatory	*TNF*	*IL1* *β*	*IL6*	*AKT1*	*MAPK1*	*MAPK3*	*CASP3*	*PTGS2*	*MAPK14*	*CCND1*	*CREB1*	*CDK6*	*HIF1A*

## Data Availability

Data presented in the study are included in the article; further inquiries can be directed to the corresponding authors.

## Supplementary Materials

The following supporting information can be downloaded at: https://www.mdpi.com/article/10.3390/cells13191639/s1, Figure S1: HPLC analysis of BBR; Figure S2: Effect of BBR on growth of ATCC 33591. Figure S3: Effect of BBR on ROS production. Table S1: Primer sequence.
