# Supplementary material for: Berberine Inhibits the Inflammatory Response Induced by Staphylococcus aureus Isolated from Atopic Eczema Patients via the TNF-α/Inflammation/RAGE Pathways
Source: Cells. 2024 Oct 1;13(19):1639. doi: 10.3390/cells13191639 (PMC11475634; doi:10.3390/cells13191639)
Supplement: Supplementary file 1 [file cells-13-01639-s001.zip › cells-3195953-supplementary.pptx]

## Slide 1
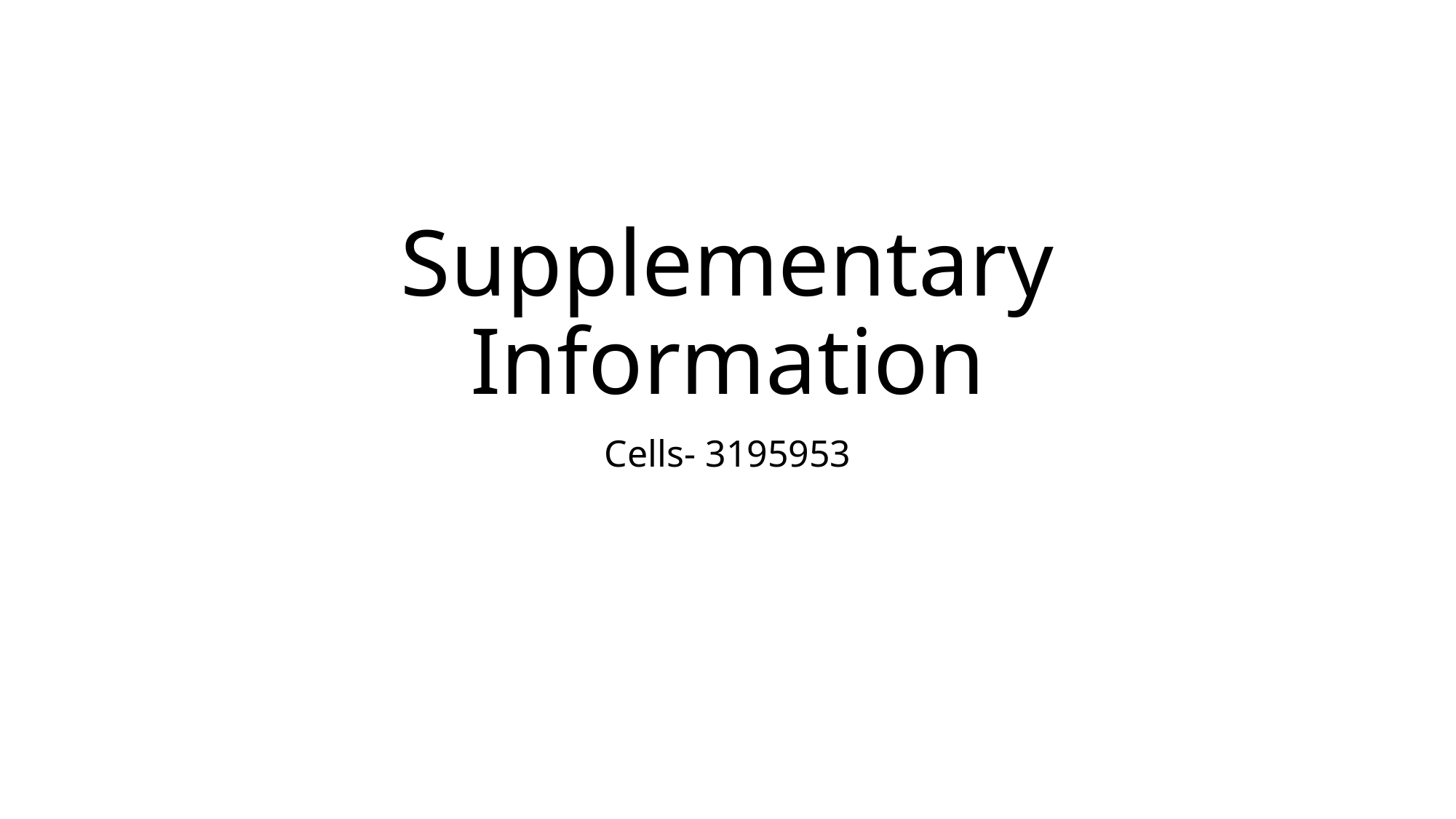

# Supplementary Information
Cells- 3195953

## Slide 2
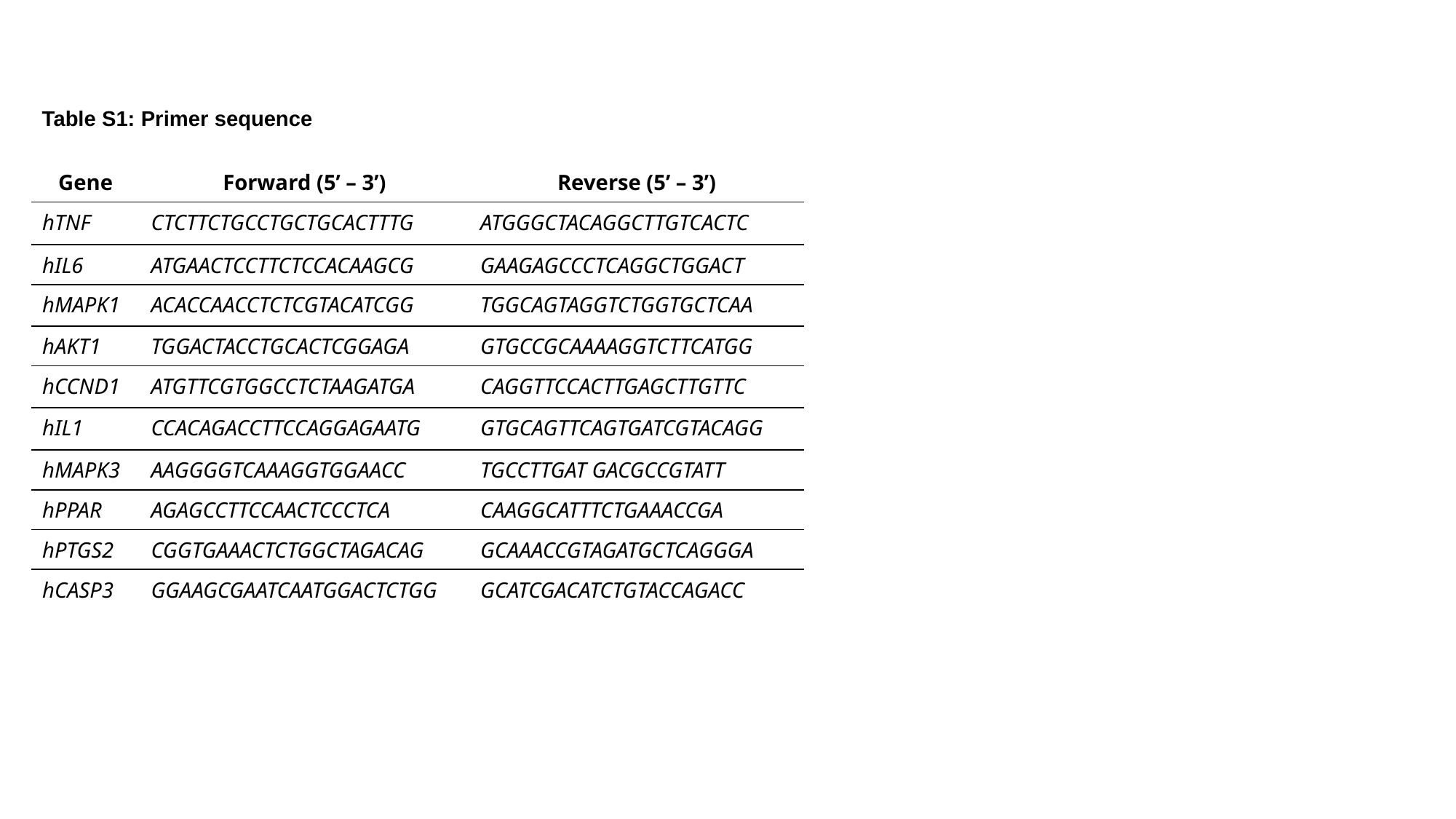

Table S1: Primer sequence

## Slide 3
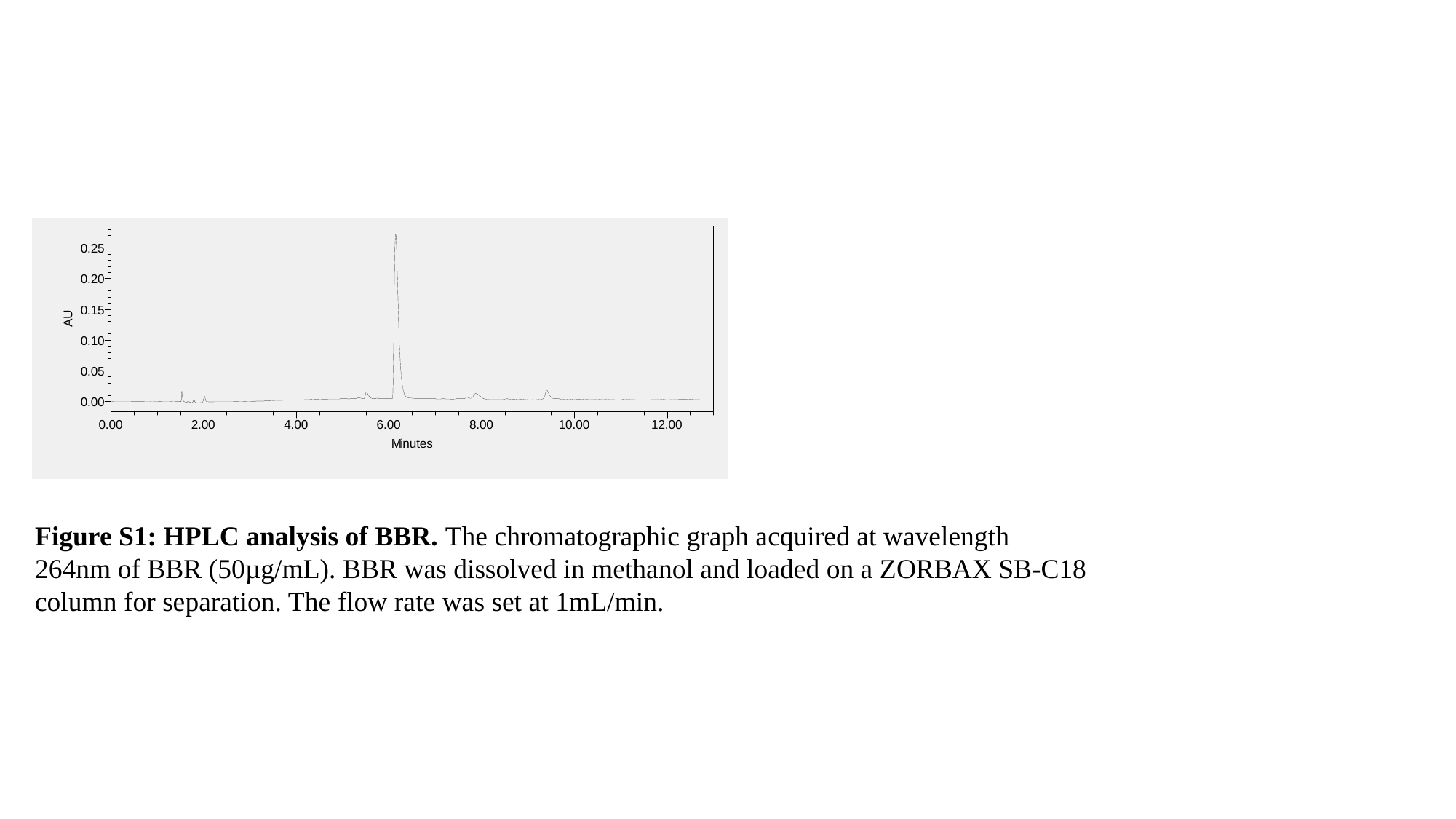

Figure S1: HPLC analysis of BBR. The chromatographic graph acquired at wavelength 264nm of BBR (50µg/mL). BBR was dissolved in methanol and loaded on a ZORBAX SB-C18 column for separation. The flow rate was set at 1mL/min.

## Slide 4
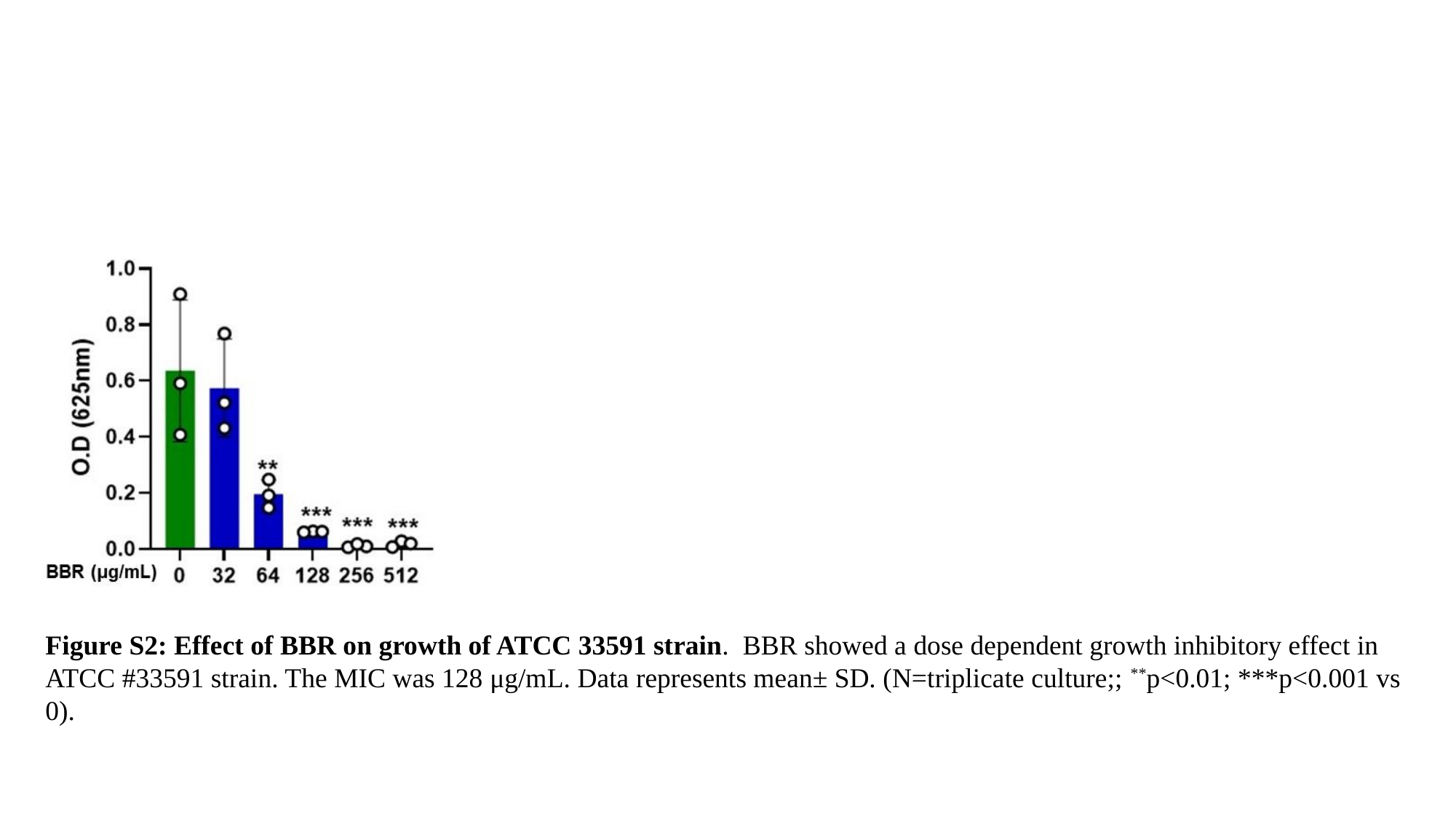

Figure S2: Effect of BBR on growth of ATCC 33591 strain. BBR showed a dose dependent growth inhibitory effect in ATCC #33591 strain. The MIC was 128 μg/mL. Data represents mean± SD. (N=triplicate culture;; **p<0.01; ***p<0.001 vs 0).

## Slide 5
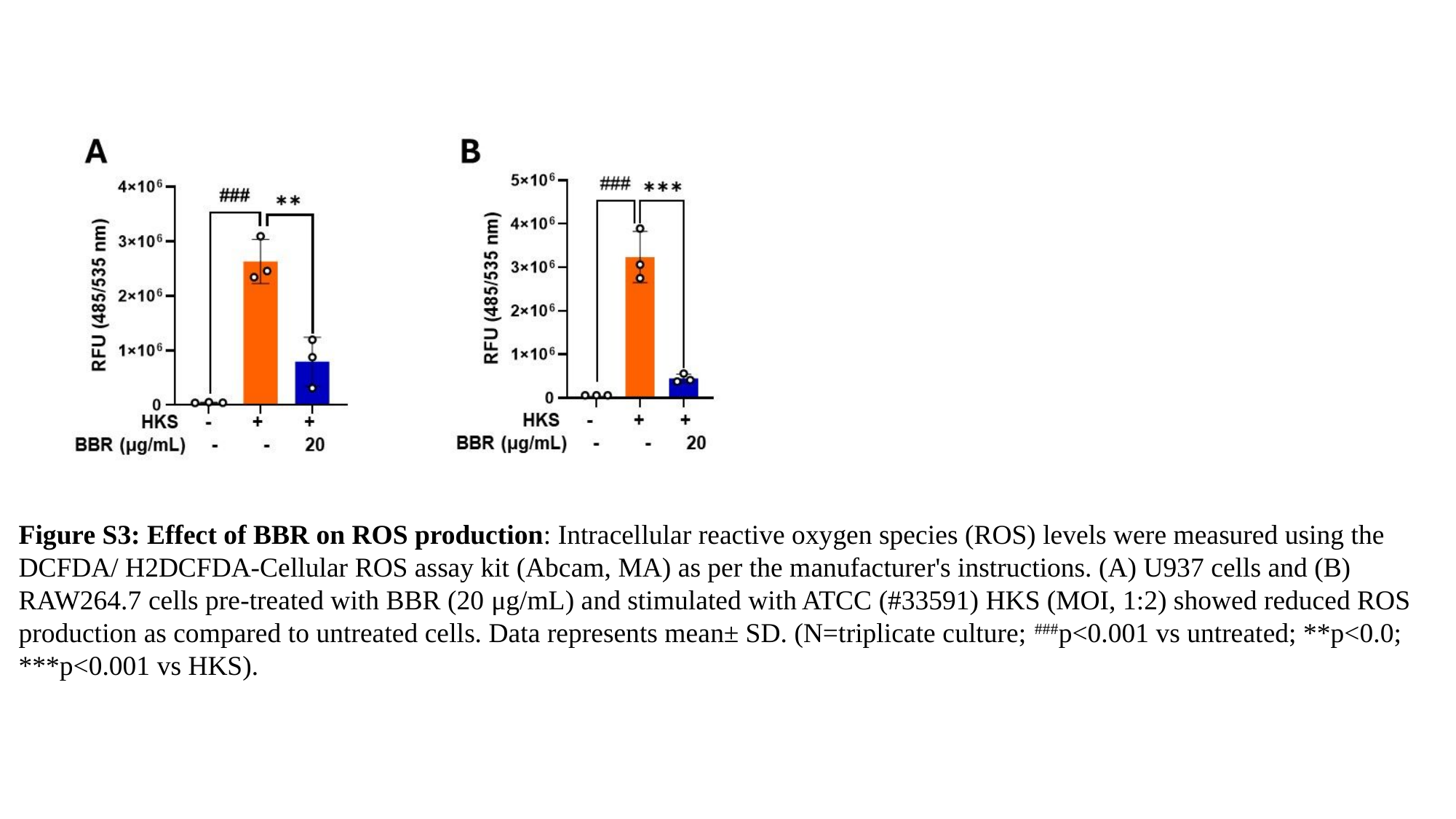

Figure S3: Effect of BBR on ROS production: Intracellular reactive oxygen species (ROS) levels were measured using the DCFDA/ H2DCFDA-Cellular ROS assay kit (Abcam, MA) as per the manufacturer's instructions. (A) U937 cells and (B) RAW264.7 cells pre-treated with BBR (20 μg/mL) and stimulated with ATCC (#33591) HKS (MOI, 1:2) showed reduced ROS production as compared to untreated cells. Data represents mean± SD. (N=triplicate culture; ###p<0.001 vs untreated; **p<0.0; ***p<0.001 vs HKS).
